# To continue or not to continue? Antipsychotic medication maintenance versus dose-reduction/discontinuation in first episode psychosis: HAMLETT, a pragmatic multicenter single-blind randomized controlled trial

**DOI:** 10.1186/s13063-019-3822-5

**Published:** 2020-02-07

**Authors:** Marieke J. H. Begemann, Ilse A. Thompson, Wim Veling, Shiral S. Gangadin, Chris N. W. Geraets, Erna van ‘t Hag, Sanne J. Müller-Kuperus, Priscilla P. Oomen, Alban E. Voppel, Mark van der Gaag, Martijn J. Kikkert, Jim Van Os, H. Filip E. Smit, Rikus H. Knegtering, Sybren Wiersma, Luyken H. Stouten, Harm J. Gijsman, Lex Wunderink, Anton B. P. Staring, Selene R. T. Veerman, Amrita G. S. Mahabir, Jörg Kurkamp, Gerdina H. M. Pijnenborg, Natalie D. Veen, Machteld Marcelis, Koen P. Grootens, Gunnar Faber, Nico J. van Beveren, Agaath Been, Truus van den Brink, Maarten Bak, Therese A. M. J. van Amelsvoort, Andrea Ruissen, Christine Blanke, Karin Groen, Lieuwe de Haan, Iris E. C. Sommer

**Affiliations:** 1 Department of Biomedical Sciences of Cells & Systems, Cognitive Neurosciences, University of Groningen, University Medical Center Groningen (UMCG), Groningen, The Netherlands; 2 Department of Psychiatry, University of Groningen, University Medical Center Groningen, Groningen, The Netherlands; 3 Department of Psychiatry, UMC Utrecht Brain Center, University Medical Center Utrecht, Utrecht, The Netherlands; 4 Parnassia Psychiatric Institute, The Hague, The Netherlands; 5 Department of Clinical Psychology, VU University, Amsterdam, The Netherlands; 6 Department of Research, Arkin Mental Health Care, Amsterdam, The Netherlands; 7 Department of Psychiatry and Neuropsychology, School for Mental Health and Neuroscience, EURON, Maastricht University Medical Center, Maastricht, The Netherlands; 8 Department of Psychosis Studies, Institute of Psychiatry, Psychology & Neuroscience, King’s College London, London, UK; 9 Department of Epidemiology and Biostatistics, Amsterdam Public Health Research Institute, VU University Medical Center, Amsterdam, The Netherlands; 10 Department of Clinical, Neuro and Developmental Psychology, Amsterdam Public Health Research Institute, VU University Medical Center, Amsterdam, The Netherlands; 11 Centre of Economic Evaluation, Trimbos Institute (Netherlands Institute of Mental Health), Utrecht, The Netherlands; 12 Lentis Research, Lentis Psychiatric Institute, Groningen, The Netherlands; 13 Rob Giel Research Center, University of Groningen, University Medical Center Groningen, Groningen, The Netherlands; 14 Early Intervention Psychosis Team, GGZ inGeest Specialized Mental Health Care, Hoofddorp, The Netherlands; 15 Centre for Early Psychosis, Parnassia Psychiatric Institute, The Hague, The Netherlands; 16 Program for Psychosis & Severe Mental Illness, Pro Persona Mental Health, Wolfheze, The Netherlands; 17 Department of Education and Research, Friesland Mental Health Care Services, Leeuwarden, The Netherlands; 18 Department ABC, Altrecht Psychiatric Institute, Utrecht, The Netherlands; 19 Community Mental Health, Mental Health Service Noord-Holland Noord, Alkmaar, The Netherlands; 20 Early Psychosis Team, GGNet, Apeldoorn, The Netherlands; 21 Center for Youth with Psychosis, Mediant ABC Twente, Enschede, The Netherlands; 22 Department of Psychotic Disorders, GGZ-Drenthe, Assen, The Netherlands; 23 GGZ Delfland, Delfland Institute for Mental Health Care, Delft, The Netherlands; 24 Institute for Mental Health Care Eindhoven (GGzE), Eindhoven, The Netherlands; 25 Reinier van Arkel Institute for Mental Health Care, ‘s Hertogenbosch, The Netherlands; 26 Radboud University Medical Centre, Nijmegen, The Netherlands; 27 Yulius, Mental Health Institute, Dordrecht, The Netherlands; 28 Antes Center for Mental Health Care, Rotterdam, The Netherlands; 29 Department of Neuroscience, Erasmus MC, Rotterdam, The Netherlands; 30 Department of Psychiatry, Erasmus MC, Rotterdam, The Netherlands; 31 Center for Developmental Disorders, Dimence Institute for Mental Health, Deventer, The Netherlands; 32 Early Intervention Team, GGZ Centraal, Amersfoort, The Netherlands; 33 Mondriaan Mental Health Care, Heerlen, The Netherlands; 34 Emergis, Kenniscentrum, Goes, The Netherlands; 35 Anoiksis, University Medical Center Utrecht, Utrecht, The Netherlands; 36 MIND Ypsilon, Organization of Relatives and Carers of People with a Vulnerability to Psychosis, The Hague, The Netherlands; 37 Department of Early Psychosis, Amsterdam UMC, Academic Medical Center, Amsterdam, The Netherlands

**Keywords:** Antipsychotic medication, first episode psychosis, Maintenance, Treatment, Discontinuation, Tapering, global functioning, Randomized controlled trial

## Abstract

**Background:**

Antipsychotic medication is effective for symptomatic treatment in schizophrenia-spectrum disorders. After symptom remission, continuation of antipsychotic treatment is associated with lower relapse rates and lower symptom severity compared to dose reduction/discontinuation. Therefore, most guidelines recommend continuation of treatment with antipsychotic medication for at least 1 year. Recently, however, these guidelines have been questioned as one study has shown that more patients achieved long-term functional remission in an early discontinuation condition—a finding that was not replicated in another recently published long-term study.

**Methods/design:**

The HAMLETT (Handling Antipsychotic Medication Long-term Evaluation of Targeted Treatment) study is a multicenter pragmatic single-blind randomized controlled trial in two parallel conditions (1:1) investigating the effects of continuation versus dose-reduction/discontinuation of antipsychotic medication after remission of a first episode of psychosis (FEP) on personal and social functioning, psychotic symptom severity, and health-related quality of life. In total 512 participants will be included, aged between 16 and 60 years, in symptomatic remission from a FEP for 3–6 months, and for whom psychosis was not associated with severe or life-threatening self-harm or violence. Recruitment will take place at 24 Dutch sites. Patients are randomized (1:1) to: continuation of antipsychotic medication until at least 1 year after remission (original dose allowing a maximum reduction of 25%, or another antipsychotic drug in similar dose range); or gradual dose reduction till eventual discontinuation of antipsychotics according to a tapering schedule. If signs of relapse occur in this arm, medication dose can be increased again. Measurements are conducted at baseline, at 3, and 6 months post-baseline, and yearly during a follow-up period of 4 years.

**Discussion:**

The HAMLETT study will offer evidence to guide patients and clinicians regarding questions concerning optimal treatment duration and when to taper off medication after remission of a FEP. Moreover, it may provide patient characteristics associated with safe dose reduction with a minimal risk of relapse.

**Trial status:**

Protocol version 1.3, October 2018. The study is active and currently recruiting patients (since September 2017), with the first 200 participants by the end of 2019. We anticipate completing recruitment in 2022 and final assessments (including follow-up 3.5 years after phase one) in 2026.

**Trial registration:**

European Clinical Trials Database, EudraCT number 2017-002406-12. Registered 7 June 2017.

## Background

Antipsychotic medication is effective in diminishing severity of psychotic symptoms and in reducing the risk for psychotic relapse [1]. Most current guidelines state that individuals with a first episode of psychosis (FEP) should be offered antipsychotic medication for at least 1 year after remission of psychotic symptoms (National Institute for Clinical Excellence guidelines, 2014 (UK) [2]; Early Psychosis Guidelines Writing Group, 2010 (Australia) [3]; Zorgstandaard Psychose, 2017 (the Netherlands) [4]). Despite the favorable effect of antipsychotics on reducing positive symptoms, patients often have a strong wish to stop medication after a treatment duration shorter than 1 year. This wish partly reflects the negative side effects of antipsychotic medication, such as weight gain, anhedonia, sedation, sexual dysfunction, and parkinsonism [5]. Therefore, in day-to-day practice, patients, their relatives, as well as clinicians face the question: to continue or not to continue?

### Discontinuation or maintenance therapy: relapse rates

A meta-analysis including 65 trials has shown that maintenance therapy of antipsychotic medication after remission reduced the risk of relapse more than twofold (i.e., 27% relapse rate with maintenance treatment versus 64% relapse in a year without medication) [6]. More recently, a systematic review conducted by Karson and colleagues [7] addressed the long-term effects and also found that continuation of antipsychotic medication was more effective than treatment discontinuation or intermittent/guided discontinuation in preventing relapse. However, it is important to note that most of the summarized trials were not designed to test continuation versus discontinuation. Alvarez-Jimenez and colleagues [8] specifically reviewed trials that randomized FEP patients to either dose reduction/discontinuation or maintenance treatment. They included eight randomized controlled trials; follow-up time of the included studies varied between 1 and 2 years. The overall relapse rate was higher in the dose reduction/discontinuation groups compared to maintenance treatment. This review was recently updated by Thompson and colleagues [9] including one extra study [10]; their conclusions were similar as relapse rates were higher in the discontinuation group (53%) versus the maintenance treatment group (19%) after a follow-up period of 1 to 2 years.

To date, only a few randomized trials have been conducted over a longer follow-up period of more than 2 years. Wunderink and colleagues [11] were the first to show long-term positive effects of early-course discontinuation of antipsychotic treatment, which may shed a different light on previous studies with a shorter duration. While they observed that the relapse rates were initially higher for the discontinuation strategy (43%) versus the maintenance condition (21%) after a follow-up period of 2 years, the relapse rates were equal after 3 years follow-up [12]. Moreover, after 7 years, dose reduction/discontinuation patients showed higher functional recovery rates versus patients following maintenance treatment. Recently, Hui et al. [13] reported on their 10-year follow-up study. Notably, they found higher rates of poor long-term clinical outcome in the discontinuation group (39%) compared to the maintenance treatment group (21%). Moreover, relapse was a significant predictor of 10-year clinical outcome.

### Discontinuation versus maintenance therapy: functional recovery

Looking beyond relapse rates, the Dutch patient organization Anoiksis argued that the decision of patients to either continue or discontinue medication should mainly be based on its proposed impact on functioning in the main domains of everyday life (surveyed in 2017). In the short term, previous studies have found no significant difference between maintenance therapy versus dose-reduction/discontinuation on functional recovery [12, 14]. The follow-up of the Wunderink [12] study demonstrated that, after 7 years, patients in the original discontinuation condition experienced twice the functional recovery rate (40.4%) in comparison to those on maintenance treatment (17.6%) [11]. However, the recent study by Hui and colleagues [13] also investigating the effects of early discontinuation on long-term clinical outcome at 10 years was in strong contrast with the Wunderink [12] finding, as they reported a higher risk of poor clinical outcome in the discontinuation group compared to the maintenance group (respectively 39% versus 21%). Poor clinical outcome was defined by persistent positive symptoms of psychosis, treatment-resistant psychosis, or death by suicide. Finally, Tiihonen, Tanskanen, and Taipale [15] observed the risk of treatment failure after discontinuation of antipsychotic treatment in a cohort of 8719 schizophrenia patients, defined as psychiatric re-hospitalization or death. The lowest risk of treatment failure was observed in patients treated with antipsychotic drugs continuously, followed by patients who discontinued medication immediately after discharge from hospital treatment. Notably, when antipsychotic drugs were discontinued at a later stage, the risk of treatment failure was even more increased (possibly explained by changes in dopamine sensitivity or by confounding by indication).

### Discontinuation versus maintenance therapy: emotional and cognitive functioning

In the context of functional recovery, the impact of (dis)continuation on emotional and cognitive functioning needs to be evaluated. Blockade of the dopamine D_2_ receptors, the main mediator of efficacy of antipsychotic medication [16], can produce adverse subjective experiences or neuroleptic dysphoria [17–19], encompassing a variety of unpleasant subjective changes in arousal, mood, thinking, and motivation [20]. Severity of these mental adverse effects depends on individual variability of sensitivity and proportion of D_2_ receptors blocked. Individuals with lower baseline dopamine function are at increased risk for dysphoric responses during treatment with dopaminergic blocking drugs [20]. With regard to dosage of antipsychotic medication, most mental adverse effects occur at D_2_ receptor occupancy higher than 65–70% [17]. In addition to dysphoria, dopamine blockade may reduce functioning by exerting negative effects on cognition. Dopamine plays an important role in learning and motivation, as it enables associative learning, especially of aversive stimuli [21]. Approximately 50% of men and up to 70% of women report difficulty in concentrating or tiredness with the use of antipsychotic medication [22]. Blockade of this system reduces the cognitive capacity to learn new associations, which may hinder study or work [23]. Blockade of the mesolimbic reward system also reduces motivation and drive, which can be expected to hamper professional and social success [24].

Mental and cognitive adverse effects associated with higher doses may explain why functional recovery can improve when patients reduce or discontinue the dose of their antipsychotic medication. Despite these theoretical expectations, cognitive improvement after continuation of treatment as compared to dose reduction/discontinuation in patients with FEP has been reported by seven studies, with a sustained effect for up to 2 years [7]. This may be explained by the deleterious effects of recurrent psychotic episodes on cognition [25]. While dopamine blockade may be aversive for mood and cognition, the effect of a psychotic relapse on both these domains may be even more substantial, leading to the relatively larger improvement when maintaining on antipsychotic treatment. However, an add-on study to the Wunderink et al. [12] trial found that dose reduction/discontinuation was associated with more improvements in neurocognitive functioning in FEP patients 5 months after receiving remission, compared to those maintaining on second-generation antipsychotics [26]. Importantly, long-term effects are still unclear, and more knowledge is needed on how maintenance therapy and dose reduction/discontinuation affect emotional and cognitive functioning in FEP patients.

### The current HAMLETT study

Taken together, previous trials comparing dose reduction/discontinuation versus treatment maintenance have indicated that continuation of antipsychotic medication reduces the risk of psychotic relapse in remitted FEP patients. However, harmful effects may also be associated with maintenance treatment [27] and two studies with long-term follow-up have shown contradictory findings [12, 13]. This makes it difficult to determine best practices based on the current literature. Patients, their relatives, as well as clinicians need to know whether dose reduction is a beneficial option for them after remission of psychosis or not, particularly in terms of global functioning, thereby going beyond symptomatic remission. This knowledge is needed to inform decisions concerning when to taper off antipsychotic medication and to evaluate which factors moderate safe dose reduction. Here we will describe the rationale, design, and methods of a pragmatic single blind randomized controlled trial in the Netherlands: the HAMLETT study (Handling Antipsychotic Medication: Long-term Evaluation of Targeted Treatment).

## Methods/design

This paper is written in line with the SPIRIT (Standard Protocol Items: Recommendations for Interventional Trials) 2013 explanation and elaboration [28], see Additional file 2.

### Aim and objectives

The aim of the HAMLETT study is to investigate whether long-term (i.e., 4 years) functional and symptomatic recovery of patients remitted from a FEP is improved when they gradually reduce their antipsychotic medication 3 to 6 months after remission of psychotic symptoms, or when they continue to use medication for at least 1 year after remission. The following research questions will be addressed:

1. Do patients in the dose-reduction/discontinuation condition achieve a higher level of global functioning compared to the maintenance condition?
2. Does subjective wellbeing, somatic health (including metabolic syndrome), relapse rates, and hospitalizations differ between the dose-reduction/discontinuation condition and the continuation condition?
3. Do rates of self-harm (aggressive incidents, suicide attempts, and suicide) differ between the maintenance treatment and the dose-reduction/discontinuation condition?
4. Which baseline or follow-up characteristics are associated with successful discontinuation of antipsychotic medication?

In addition, health-economic evaluation and prognostic modeling will be conducted:

1. To assess the incremental cost-effectiveness (cost per functional recovery) of dose reduction and discontinuation relative to maintenance
2. To assess incremental cost-utility (cost per quality adjusted life year (QALY)) of dose reduction and discontinuation relative to maintenance
3. To identify patient profiles that predict the greatest net (monetary) benefits after dose reduction/discontinuation
4. To calculate budget impacts of scaling up dose reduction and discontinuation strategies, should these strategies be proven acceptable, effective, and cost-effective

### Trial design and setting

HAMLETT is a pragmatic single-blind randomized controlled trial of continuation versus discontinuation/dose reduction of antipsychotic medication in patients remitted after a FEP. To provide clear, clinically relevant guidance for clinicians and patients on short- and long-term benefits and disadvantages of continuation or discontinuation/dose reduction of antipsychotic treatment, the study population and their treatment should resemble the general clinical situation as much as possible. Recruitment will take place at 24 Dutch sites.

The study is divided in two phases: (1) an experimental phase of 6 months, (2) a follow-up phase of 3.5 years. The experimental phase consists of a screening visit (− 3 to 0 months before participating), a baseline visit, a midterm visit (at 3 months post-baseline), and a close-out visit (6 months post-baseline). The follow-up phase consists of four visits (i.e., at 12, 24, 36, and 48 months post baseline). Study examinations scheduled in the course of the study are listed in Table 1 (these are described in more detail in Appendix 2).

**Table 1:**
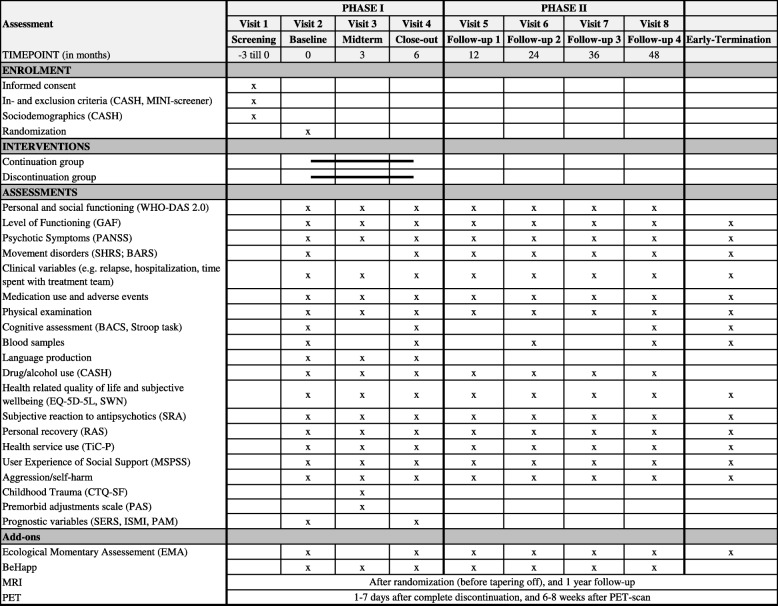
Overview of assessments during the trial

*Abbreviations*: *BACS* Brief Assessment of Cognition in Schizophrenia, *BARS* Barnes Akathisia Rating Scale, *BeHapp* Smartphone application, *CASH* Comprehensive Assessment of Symptoms and History, *CTQ-SF* Childhood Trauma Questionnaire-Short Form Multidimensional Scale of Perceived Social Support, *EMA* Ecological Momentary Assessments, *GAF* Global Assessment of Functioning, *MINI-Screener* Mini-International Neuropsychiatric Interview Screener, *MSPSS* Multidimensional Scale of Perceived Social Support, *PANSS* Positive And Negative Symptom Scale, *SHRS* St. Hans Rating Scale, *WHO-DAS 2.0* World Health Organisation Disability Assessment Schedule

### Study population and eligibility criteria

#### Study population

A total of 512 patients will be included with a first episode of schizophrenia, schizoaffective disorder, schizophreniform disorder, brief psychotic disorder, delusional disorder, substance/medication-induced psychotic disorder, or those classified as Unspecified Schizophrenia Spectrum and Other Psychotic Disorders (DSM-5, or as described in the International Classification of Diseases-10), who are in remission for 3–6 months. Patients will be recruited from both inpatient and outpatient settings in 24 health care centers throughout the Netherlands. Randomization (1:1) will be stratified according to the collaborating centers (see Appendix 1 for a list of study sites and health care centers).

##### Inclusion criteria

1. The participant has had a first episode of psychosis and uses antipsychotic medication.
2. Psychotic symptoms are in remission for 3-6 months.
3. Age 16-55 years.
4. The participant understands the study and is able to provide written informed consent.
5. HAMLETT is the only medical-scientific medication study in which the patient participates.
6. Sufficient knowledge and ability of the Dutch language.

#### Exclusion criteria

1. Dangerous or harmful behavior (i.e., behavior with a risk of severe physical injury, or actual physical injury inflicted, to self or others) occurred during FEP
2. Coercive treatment with antipsychotic medication during FEP (based on a judicial ruling)

#### Patient withdrawal

Subjects can leave the study at any time for any reason if they wish to do so, without any consequences. The clinician or investigator can decide to withdraw a subject from the study for urgent medical reasons.

#### Interventions

##### Continuation condition

Patients in the continuation condition are treated according to Dutch guidelines [4, 29], which recommend at least 1-year continuation after remission. During this year, medication will be kept within the same range, allowing a 25% dose reduction; increase of dosage is not restricted. After that first year, a shared decision is made for further continuation or gradual discontinuation based on the patient’s motivation and the clinical situation (in case of discontinuation, the tapering schedule as described below can be used). Patients and their treatment team may diverge from this regimen for several reasons, such as intolerable side effects, insufficient efficacy, or the wishes of the patient. In such cases, the patients will remain in the study.

##### Discontinuation/dose reduction condition

Discontinuation schedules have been prepared by the study team for common antipsychotic drugs available in the Netherlands (including haloperidol, risperidone, quetiapine, olanzapine, clozapine, and aripiprazole; Additional file 1). Discontinuation schedules were constructed on the following principles: smooth and gradual regular lowering of the serum levels of antipsychotic medication. Since we could not use tapering strips, we needed to diminish antipsychotic medication depending on availability of different dosages and the possibility to divide tablets. Treating physicians prescribe the tapering schedule that fits the patient’s type and dose of baseline medication, yet details can be tailored in collaboration with the patient and important relatives. When dose reduction is successful, patients can discontinue their medication completely. Duration of the discontinuation period depends on the starting dose (see Additional file 1). The average duration until complete discontinuation is 3 months.

**Fig. 1:**
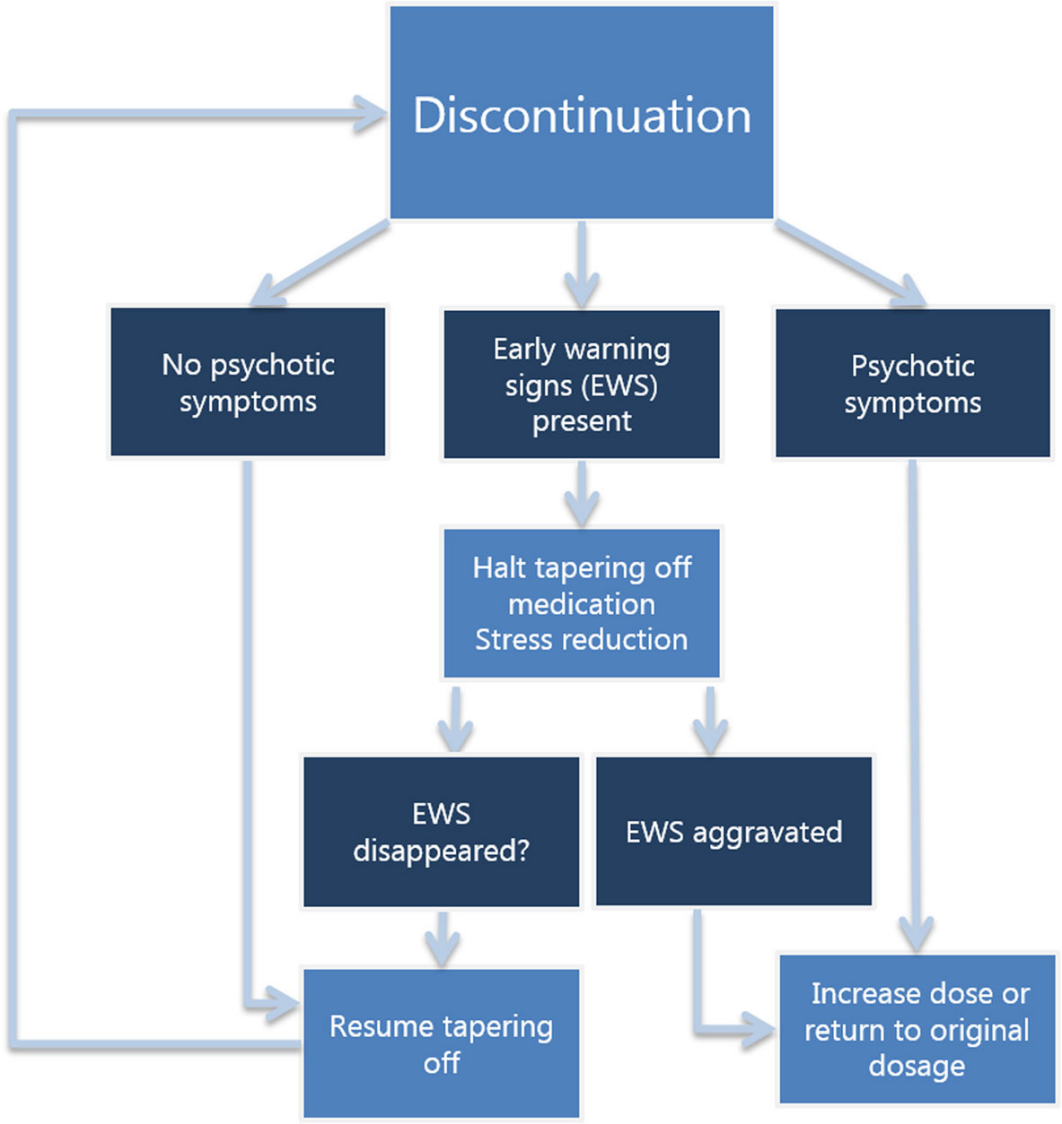
An overview of the procedure when early warning signs or psychotic symptoms reappear while tapering off medication. *EWS* early warning signs

In a letter for the treating physician, the study team provides recommendations on discontinuation schedules for the various antipsychotics used (Additional file 1) and provides a diary to be used by participants during the tapering process, providing practical advice and a questionnaire focusing on possible early warning signs for psychotic relapse. A signaling plan describing early warning signs and a plan of action is made with the treating physician prior to tapering off medication. Patients can find early warning signs (e.g., social withdrawal, sleep disturbances) in a booklet provided to the patient at the beginning of the study, and they are also noted by the patient/caregiver/family/relatives of the patient. Patients and their treatment team may opt to halt discontinuation at any time or dose when (subclinical) symptoms reappear, in which case participants will remain in the study, even though further discontinuation is not deemed possible. In case early warning signs occur, further tapering off of antipsychotic medication will be halted until early warning signs disappear. Stress reduction will be advised. When early warning signs disappear, tapering off antipsychotic medication can be resumed. When early warning signs become more severe, the dosage of antipsychotic medication will be increased to one level higher (in other words, back to the former step) of the tapering off scheme. When psychotic symptoms occur, treatment with antipsychotic medication will be restarted in the dose that patients used when the first symptomatic remission occurred. See Fig. 1 for an overview.

### Measures

#### Primary outcome

Personal and social functioning will be evaluated using the WHO-DAS 2.0 disability scale [30]. This questionnaire will be administered as an interview and consists of 36 items covering six domains of functioning in everyday life: cognition (understanding and communicating), mobility (moving and getting around), self-care (hygiene, dressing, eating, and staying alone), getting along (interacting with other people), life activities (domestic responsibilities, leisure, work, and school), and participation (joining in community activities).

#### Secondary outcomes—cognitive measures

Neurocognitive functioning will be assessed with the Brief Assessment of Cognition in Schizophrenia [31] (BACS). The BACS consists of the following domains:

1. Verbal memory: List learning
2. Working memory: Digit sequencing task
3. Motor speed: Token motor task
4. Verbal fluency: Category instances
5. Verbal fluency: Controlled oral work association test
6. Attention and speed of information processing: Symbol coding
7. Executive functions: Tower of London

#### Clinical outcomes

1. General functioning will also be evaluated using the Global Assessment of Functioning scale [32] (GAF).
2. Psychotic symptom severity will be measured with the Positive and Negative Symptom Scale [33] (PANSS).
3. Quality adjusted life years (QALYs) will be measured using the EuroQoL [34] (EQ-5D-5 L).
4. The presence and severity of movement disorders will be evaluated using St. Hans Rating Scale [35] (SHRS) and Barnes Akathisia Rating Scale [36] (BARS).
5. Personal recovery with a special focus on hope and self-determination will be assessed using the Recovery Assessment Scale [37] (RAS).
6. Other study parameters are psychotic relapse, rehospitalization, somatic health, obesity, parkinsonian side effects, depressed mood or anxiety, clinical variables (e.g., medication use, time spent with treatment team, premorbid adjustment, side effects), somatic health will be evaluated by measuring weight, height, blood pressure, waist circumference, and body mass index (BMI), and safety data will be evaluated by comparing incidences (number and percentage of subjects) of key serious adverse events (SAEs) between both groups (e.g., relapse and hospitalizations; Appendix 3).

#### Baseline characteristics

1. Diagnostic information and (socio)demographics will be collected using the Comprehensive Assessment of Symptoms and History [38] (CASH).
2. During each occasion, the following blood levels were determined: high density lipoprotein cholesterol (HDL-C) and fasting glucose, cholesterol, LDL, C-reactive protein (CRP), blood levels of the antipsychotic used. DNA isolation and aliquotation of the serum will also be done.
3. Experience of childhood trauma will be assessed at baseline using the Childhood Trauma Questionnaire–Short Form [39] (CTQ-SF).

#### Speech production

Antipsychotic medication is known to interact with receptors in language-related areas in the brain [40]. Therefore, antipsychotics are likely to influence language production in patients with a psychotic disorder. By analyzing phonetic, syntactic, and semantic aspects of recorded spoken language using a semi-structured interview at different time points during the study, we aim to analyze the effect of antipsychotic medication on language production.

### Optional studies

#### Ecological momentary assessments and BeHapp

Two embedded ecological momentary assessment (EMA) [41] studies which use smartphone diary apps will be performed. In study 1, 88 patients be will assessed ten times a day at semi-random moments during 7 days to measure momentary positive/negative affect, paranoia, hallucinations, social company, and social functioning and activities. EMA will be completed at baseline, 6 months, and 1, 2, 3, and 4 years follow-up. For study 2, a sub-sample of 30 patients will complete an intensive series of EMA during 16 consecutive weeks in order to analyze within individuals to which degree early changes in the dynamic system of mental states predict future clinical change. Furthermore, we also ask participants to install the ‘BeHapp’ smartphone application [42], in which several aspects of daily life concerning social behavior will be measured passively. The application will continuously monitor frequency and duration of smartphone usage (but not content), as well as information on Bluetooth connections, WiFi, and GPS locations. For a detailed description, see Appendix 3.

#### Magnetic resonance imaging outcome measures

Differences in brain volume between continuation and discontinuation of antipsychotic medication will be investigated by means of structural Magnetic Resonance Imaging (sMRI). Specifically, we will scan 150 patients at baseline, before (dis)continuation and after 12-month follow-up. We will evaluate the effects of antipsychotics on total brain volume and on specific structures such as hippocampus, thalamus, caudate, and parietal and prefrontal cortex, including effects of type of medication and gender. Potential brain volume loss within individuals will be investigated by comparing the two scans with a 12-month follow-up.

#### Positron emission tomography

Discontinuation of antipsychotic medication after the use of these drugs for several months may render patients especially vulnerable to relapse. The potential mechanism behind this vulnerability could be increased density of postsynaptic dopamine D_2_ receptors in the striatum. We will investigate the presence of dopaminergic abnormalities, as measured with [11C] raclopride, in relation to antipsychotic medication discontinuation in 30 patients remitted after a FEP. We will scan patients 1–7 days after discontinuation and 6–8 weeks after the first scan.

#### Resource use

Patients’ health care usage and productivity losses will be measured with the Trimbos and iMTA Cost questionnaire associated with Psychiatric illness [43] (TiC-P), which is the most commonly used health service receipt interview in the Netherlands. This is required to compute heath care costs (including intervention costs), the patients’ out of pocket costs for making round trips to health services, the opportunity costs of relatives and friends when offering care to the patient, and to assess the costs stemming from productivity losses when patients are on sick leave (absenteeism) and when they cut back on work while at work (presenteeism).

#### Safety measures

After inclusion in the study, a personal patient profile in which individual early warning signs of impending relapse are described will be created. These signs are the individual prodromal signs a patient experienced before their first psychotic episode. Patients and relatives will be instructed to contact professional caregivers in case of occurrence of early warning signs. Treatment will be modified in case of occurrence of early warning signs or other indications of clinical worsening. Documentation of occurrence and severity of signs and symptoms and treatment modification will be assessed during each visit. Patients and their involved relatives will be advised on the tapering off or continuation scheme (depending on the condition and depending on occurrence of early warning signs or psychotic relapse). Adverse events (AEs) are defined as any substantial undesirable experience occurring to a subject during the study (including a psychotic relapse without hospitalization), whether or not considered related to treatment allocation. All AEs and SAEs reported spontaneously by the subject or observed by the clinician or research staff will be recorded, according to the protocol, in the electronic case report form (eCRF).

### Sample size

This study will use WHO-DAS 2.0 [30] personal and social functioning as a continuous primary outcome and is powered to detect a standardized mean effect of at least *d* = 0.33 (minimal effect deemed clinically relevant by Lipsey and Wilson [44]). We assume a clustering effect in the data corresponding to an intra-class correlation coefficient of 0.05. Tests will be conducted with alpha = 0.05 (two-sided) and a power (1-beta) = 0.80. This requires 230 participants per arm. Given the long follow-up, we expect dropout. Although an intention-to-treat analysis is robust against moderate dropout, we aim to include an extra 10% to compensate for dropout. Therefore, we aim to randomize 230/(1 − 0.10) = 256 per arm, or 512 patients in total.

### Recruitment and allocation

#### Recruitment

In total, 24 different specialized health care centers collaborate in the HAMLETT study. Each participating center has a principal investigator (PI) to promote and implement the study within their organization. Each site also has a (part-time) dedicated includer (DI; i.e., who preferably is a member of the clinical staff, for instance a nurse) with the task of facilitating inclusion and assisting clinicians by selecting and inviting potential participants.

#### Allocation

The randomization will take place after the baseline visit and is conducted by unblinded members of the research team. A web-based application will be used (random.org/sealedenvelipe.com), randomization is stratified for treatment according to the collaborating centers, with a 1:1 allocation ratio. The randomization outcome is communicated directly to the treating physician, together with a suggestion for discontinuation/dose reduction schedule (Table 1) if their patient is randomized to this group. The general practitioner and pharmacist of the patient are also informed.

#### Blinding

This study will be single-blind: only the assessor who performs the assessments and conducts the interviews is blind for the treatment condition of the patient. When blinding is broken, for example, because a patient communicates about his medication use or discontinuation, the assessor is replaced by another rater who is still blind. Clinicians and patients are not blinded.

### Data collection methods and management

Patient visits and examinations specified per visit can be found in Table 1; these are described in more detail in Appendix 2. Participants in the HAMLETT study will receive a gift voucher at each visit, in each study condition (as approved by the ethics committee of the University Medical Center Groningen). Data collection forms are on paper and entered into an eCRF. To ensure data quality, assessors are comprehensively informed and trained regarding Good Clinical Practice (GCP). Experts train users in the proper conduct of the WHO-DAS-2.0 [30], BACS [32], PANSS [33], CASH [38] interview, movement disorder scales (SHRS [35], BARS [36]), and cognitive testing. In addition, the team of assessors have biannual meetings every 6 months during which inter-rater reliability is assessed, new assessors are trained, supervision is given, and protocol adherence is checked.

Privacy laws and regulations will be adhered to during the length of the study. The collection and processing of participants’ personal information will be limited to what is necessary to ensure the study’s scientific practicability, the evaluation of efficacy, adherence, side effects, and the investigational product’s safety. Information collected about participants during this clinical investigation will be treated confidentially. The investigator or her co-workers will collect data and transfer them without recording the patient’s name or date of birth. Instead, data will be coded with a participant identification number.

Only authorized personnel will have access to the identification key. The source documents will be kept in a locked filing cabinet with access limited to research personnel. In accordance with national laws and guidelines and the specifications of the ICH-GCP guidelines, the investigators are obligated to archive all documents pertaining to the study for the legally required time period.

The acquired data and examination results will be entered into an eCRF that is accessible via the internet. Investigators will receive personal user names and passwords for this purpose, and data will be encrypted for transfer. It will be agreed before the start of the study as to which documents serve as source documents for all data entered into the eCRF.

### Collaboration with important others

The HAMLETT study is performed in close collaboration with MIND Ypsilon, a Dutch organization of relatives and carers of people with vulnerability to psychosis, and Anoiksis, a Dutch patient organization. When a participant is enrolled in the study, he/she is invited to bring a friend, parent, or other relative to the appointments in order to receive information as well. During the phase in which medication is tapered off, participants are encouraged to engage an important other in this process (this can be a parent, partner, sibling, or close friend). Both the participant and his/her close associate are given a booklet which contains information about potential risks and gains associated with tapering off medication. This also includes a list of questions to assess early warning signs and signs of relapse. Telephone numbers are supplied to indicate how to reach both the treatment team and the HAMLETT study team.

### Statistical methods

#### Hypothesis testing

Research questions 1 to 4 will be tested using generalized linear mixed modeling for continuous outcomes (WHO-DAS 2.0 functioning as a continuous outcome), logistic models for binary outcomes (WHO-DAS 2.0 recovery), and Poisson models for tallies (0, 1, …, N) of psychotic relapses and hospitalizations. The models will take into account the clustered data structure of repeated measures within each patient, and patients being nested in treatment centers. Data will be analyzed according to the intention to treat principle. These analyses will be conducted for both primary outcome and secondary outcomes. The tests will be conducted at α ≤ 0.05 (two-tailed), and reported as stipulated by the CONSORT statement.

#### Health-economic evaluation

A cost-utility analysis (CUA) and cost-effectiveness analysis (CEA) will be conducted alongside the study with quality adjusted life years (QALYs) gains and WHO-DAS 2.0 functional recovery as the main outcomes, respectively. Costs will be computed by multiplication of health care units (visits, sessions, contacts, hospital days) by their appropriate standard cost price. Missing cost and outcome data will be imputed using multiple imputation for intention-to-treat (ITT) analysis. Since the study’s follow-up measurements exceed 1 year, both costs and effects will be discounted by 4% and 1.5%, respectively. Cumulative costs and QALY health gains over the study’s follow-up period will be computed with the area under the curve method. The incremental cost-effectiveness ratio (ICER) will be computed to obtain the incremental costs per WHO-DAS 2.0 functional recovery and the incremental costs per EQ-5D-5 L QALY gained. Stochastic uncertainty will be handled using 2500 non-parametric bootstraps and by plotting the simulated ICERs on the ICER plane. For decision-making purposes, the ICER acceptability curve will be graphed for various willingness-to-pay (WTP) ceilings for making judgments whether the dose-reduction/discontinuation intervention offers good value for money relative to maintenance. One-way sensitivity analyses directed at uncertainty in the main cost drivers (e.g., costs of hospital re-admissions after psychotic relapse) and outcomes (e.g., under different imputations) will be performed to assess the robustness of our findings. Both the analysis and reporting of the research findings will conform to the CHEERS statement [45, 46].

#### Prognostic modeling

Prognostic modeling will be used to identify patient characteristics that predict (1) successful WHO-DAS 2.0 functional recovery, (2) successful discontinuation without psychotic relapses, and (3) greater net-benefits (QALY gains valued in euros minus health care costs). Prognostic modeling will be conducted in R with a suite of models (logistic regression, K-nearest neighbors, classification tree, random forests, gradient boosting, and support vector machine) and will be driven by the following expectations:

1. Patients with longer duration of untreated psychosis, comorbid drug abuse, male gender, lower education and earlier onset of psychosis will carry a poorer prognosis.
2. Dose-reduction/discontinuation will be more successful in patients who have used lower doses of medication, or have used medication with relatively low D_2_ receptor affinity (clozapine, quetiapine, and olanzapine).
3. Personal and social functioning will be superior in patients who participated in psychosocial interventions such as cognitive behavioral treatment (CBT) and individual placement and support (IPS).
4. Psychotic relapse rates after discontinuation will be lower in patients who received CBT and IPS.

In short, these analyses will address the question of what works best for whom, and may support treatment decisions such as which patients are best referred to dose-reduction and discontinuation.

#### Interim analysis

Interim analyses are planned to assess if one of the trial’s conditions (either discontinuation or continuation) is associated with markedly inferior outcomes. Interim analyses will be performed after 1 and 3 years by an independent statistician. Dr. Klaas Wardenaar (University Medical Center Groningen, Faculty of Medical Sciences, the Netherlands) kindly agreed to assume this role. The interim analyses will be conducted for the primary efficacy end point of the study obtained from patients in the target population. The statistical analyses will be carried out at the two-sided overall alpha-level of 0.05. The type I error boundaries for statistical significance will be adjusted for multiple comparisons (i.e., total number of analyses = 3). A design-based error spending function using the O’Brien-Fleming boundaries will be applied [47]. The O’Brien-Fleming plan allocates the alpha error conservatively across the interim and final analyses in the study. At the first interim analysis, a two-sided *p* value will be declared significant if it is less than 0.0021; at the second interim analysis, the respective alpha error boundary will be 0.0105. At the final analysis, the two-sided *p* value will be declared significant if it is less than 0.025. Based on the outcome at the interim stage (i.e., if *p* < 0.0021 or *p* < 0.0105, for the two interim analyses, respectively), the study can be stopped for overwhelming evidence of group difference.

### Data monitoring

#### Medical ethical review board

Ethics approval covering all participating sites was obtained from the research and ethics committee of the University Medical Center Groningen, the Netherlands, protocol number NL 62202.042.17.

#### Declaration of Helsinki

The study will be conducted in accordance with this protocol as well as the principles of the Declaration of Helsinki (64th WMA general assembly; October 2013). Information collected about participants during this clinical investigation will be treated confidentially.

#### Patient safety

The study team can at all times be contacted at the telephone number provided on the contact card and letters that the patients receive during the study. The patients’ day-to-day care is the responsibility of the treating physician.

The sponsor/investigator has a liability insurance which is in accordance with article 7, subsection 6 of the WMO. The sponsor (also) has insurance for participants in accordance with the legal requirements in the Netherlands (Article 7 WMO and the Measure regarding Compulsory Insurance for Clinical Research in Humans of 23 June 2003). This insurance provides cover for damage to research subjects through injury or death caused by the study.

1. €650,000.-- (i.e., four hundred and fifty thousand euro) for death or injury for each subject who participates in the research;
2. €5,000,000.-- (i.e., three million five hundred thousand euro) for death or injury for all subjects who participate in the research;
3. €7,500,000.-- (i.e., five million euro) for the total damage incurred by the organization for all damage disclosed by scientific research for the Sponsor as ‘verrichter’ in the meaning of said Act in each year of insurance coverage.

The insurance applies to damage that becomes apparent during the study or within 4 years after the end of the study.

#### Amendments

A “substantial amendment” is defined as an amendment to the terms of the ERB application, or to the protocol or any other supporting documentation, that is likely to affect to a significant degree:

- The safety or physical or mental integrity of the subjects of the trial
- The scientific value of the trial
- The conduct or management of the trial, or
- The quality or safety of any intervention used in the trial

All substantial amendments will be submitted for approval to the ERB and to the competent authority. For non-substantial amendments, only a notification will be sent to the accredited ERB, which will be recorded and filed by the sponsor.

#### Public disclosure and publication policy

The results of the study will be submitted for publication in an international peer-reviewed journal adhering to applicable privacy laws and regulations. Publication strategy will be determined by the principal investigator. No treatment group information will be made available until after study completion.

## Discussion

The HAMLETT study investigates the effects of continuation versusdose-reduction/discontinuation of antipsychotic medication after remission of FEP on personal and social functioning, psychotic symptom severity, health-related quality of life, and cognitive functioning, amongst a range of other relevant outcomes. Many studies comparing maintenance treatment with dose reduction/discontinuation have consistently shown that dose reduction/discontinuation increases the risk of psychotic relapse in remitted FEP patients [7–9]. Notably, relapse may be associated with antipsychotic treatment resistance in schizophrenia. A recent study by Takeuchi et al. [48] suggests a reduced and/or delayed antipsychotic treatment response in the face of relapse following effective treatment of first episode schizophrenia. Yet, the first study with a long follow-up time by Wunderink and colleagues reported better outcomes after 7 years with early discontinuation in terms of symptomatic and functional remission compared to maintenance treatment [11]. The recent study conducted by Hui and colleagues [13] could not replicate this finding, as they found a higher risk of poor clinical outcome in the discontinuation group compared to the maintenance group when evaluating long-term clinical outcome at 10 years. This underlines the importance of additional long-term cohorts to systematically investigate the effects of the two strategies on different outcomes. HAMLETT is a long-term, well-powered study which is conducted and supported in the majority of Dutch early psychosis treatment units.

Currently, similar trials are being conducted: the TAILOR trial [49] (Denmark), the RADAR study (research into antipsychotic discontinuation and reduction; UK), the reduce trial [50] (Australia), and “A Guided Dose Reduction Trial for Patients with Remitted Psychosis” [51] (Taiwan).

### Strengths and limitations of a pragmatic trial

The HAMLETT study is aimed to be the largest randomized controlled trial yet reported that investigates the effects of maintenance treatment versus dose reduction/discontinuation for FEP. HAMLETT is a pragmatic trial, with the population and their treatment resembling the general clinical situation as much as possible to increase ecological validity and also to pave the way to future implementation. We opted for this design as this study aims to provide clear guidance for clinicians and patients on short- and long-term benefits and disadvantages of maintenance treatment and dose-reduction/discontinuation of antipsychotic treatment. The naturalistic set-up of the study has several consequences. First, we kept the exclusion criteria as few as possible. Only when the safety of the participant is at risk will exclusion follow. Patients with, for example, comorbidity and drug- and alcohol abuse will be able to participate, which leads to a heterogeneous sample reflecting clinical practice. Second, to address the issue of selection bias, all FEP patients eligible for the study are registered by the early psychosis treatment units. Data are collected on patients who do not wish to participate in the study (e.g., reason not to participate, age, and gender). Third, patients can start the trial using any type of frequently prescribed antipsychotic drug at any dose (within safety ranges). Fourth, to prevent the average dose in the maintenance arm and the dose reduction/discontinuation arm differing too much from each other, we instruct physicians to not reduce the dose by more than 25%. Fifth, clinicians and patients are informed on the allocated condition as they should be attentive to early warning signs for relapse; researchers are blinded. Lastly, research suggests that 64% of patients discontinuing medication will relapse [6], which could be quite a substantial number in our large sample. However, we expect the relapse rate to be lower in our study as a tapering schedule is provided by which patients will gradually reduce dose over the course of 3–6 months; when this is successful, patients could discontinue completely. When early warning signs are present, the dose can be increased. This way, those who respond well to discontinuation may go on to discontinue completely. The dose of the patients that do require antipsychotic treatment can be reduced as much as possible to remain symptom free.

### Supplementary information

**Additional file 1: Table S1.** Tapering off schedules.

**Additional file 2:** SPIRIT checklist for the HAMLETT study.

## Data Availability

Not applicable as data are not yet available.

## Appendix 1

### List of study sites and collaborating centers

1. Universitair Medisch Centrum Groningen, (the “Coordinator”) / Lentis
2. Academisch Medisch Centrum Amsterdam
3. Universitair Medisch Centrum Utrecht
4. Mondriaan / Universiteit Maastricht
5. TRIMBOS instituut
6. Geestelijke Gezondheidszorg Rivierduinen
7. Arkin
8. Geestelijke Gezondheidszorg Ingeest
9. Geestelijke Gezondheidszorg Eindhoven
10. Altrecht
11. Parnassia Groep
12. Reinier van Arkel stichting
13. Geestelijke Gezondheidszorg NoordHollandNoord
14. Yulius
15. Geestelijke Gezondheidszorg Delfland
16. GGNet
17. Mediant
18. Dimence
19. Geestelijke Gezondheidszorg Drenthe
20. Delta
21. Pro Persona
22. Geestelijke Gezondheidszorg Centraal
23. Vincent van Gogh
24. Geestelijke Gezondheidszorg Breburg

## Appendix 2

### Study procedures

#### Informed consent procedure

In each of the 23 participating centers, a local dedicated includer (DI) appointed by the HAMLETT study provides information to the patient and when possible his or her relatives (the information letter including the informed consent form, and the short information brochure). The patient and his or her relatives are given two weeks (or longer if needed) to consider participation. A second appointment is made (vis a vis) in which extra questions can be answered and in which oral and written information is provided. Additional questions are answered and if the patient opts to participate, the informed consent is signed by both the patient and the DI or a member of the central study team. Patients who are thought to meet the inclusion criteria are approached, their treating physician will be notified. The treating physician decides if the patient is able to make a well-informed decision. For participants under the age of 18, both parents will be asked to write an assent in addition to the patient’s consent. Before signing of the informed consent form, no other study procedures will be executed, the DI arranges a first visit with the central study team.

#### Screening visit

Patients will be screened for eligibility to the study, after informed consent is completed. Diagnosis will be checked using the CASH [38] and using the MINI Screener (developed at the department of Psychiatry, UMC Groningen). Several demographical and clinical variables will be assessed, including date of birth, sex, educational level, zip code, living alone/together, profession, prior psychiatric disorders, date of diagnosis (CASH), premorbid functioning (PAS) and duration of untreated psychosis (retrospectively, as defined by fulfilling a score of ≥4 on one of the positive items of the PANSS during at least one whole day, until start of antipsychotic medication (adapted from Marshall et al. [52]). Medication use (including antipsychotic use, hormonal contraception and other medication) will be evaluated, in addition to side effects and subjective reactions to these medications (SRA [53], SWN [54]). Physical health will be checked in a standard physical examination (height, weight, waist circumference, Body Mass Index, blood pressure, pulse, EPS), in addition to medical history.

#### Baseline visit

At baseline, the patient will be randomized. Blood samples will be taken and substance abuse will be measured using the appropriate section of the CASH. The use of concomitant medication and medical conditions/adverse events will be recorded. In addition, the standard rating scales will be used for the first time, including primary and secondary outcomes (see below for details of these scales).

#### Study visits: V3 (3 months post-baseline) + V4 (6 months post-baseline)

The visits of phase I are scheduled three and six months after baseline. Some flexibility is allowed, as visits can be performed within a range of +/− three weeks. Visits will consist of all rating scales, blood samples and a personal interview.

#### Follow-up visits

Follow-up visits are scheduled after the discontinuation phase is completed, to assess the current status of the patient after that part of the study. There will be four annual visits, the first scheduled 6 months after completion of phase 1 (thus one year after baseline), the others with intervals of one year (+/− three weeks).

## Appendix 3

### Rating scales

- The World Health Organization’s Disability Assessment Schedule [30] (WHO-DAS 2.0). The WHO-DAS 2.0 assesses disability in individuals irrespective of diagnosis across multiple life domains. We will use the interview version consisting of 36 items.
- Side effects experienced by the use of mental health medications as assessed by the:
  - ◦ The Subjective Wellbeing under Neuroleptics [53] (SWN): self-assessment scale of the subjective experience of patients during treatment with neuroleptics. The 20-item SWN scale has an internal consistency of 0.93 and the subscale consistencies ranged from 0.70 to 0.80. Test–retest reliabilities are observed of r = 0.70. Confirmatory factor analysis replicated the presence of a higher-order factor (general well-being) and five first-order factors (mental functioning, physical functioning, social integration, emotional regulation, and self-control).
  - ◦ The Subjective Reaction on Antipsychotics [53] (SRA) is a 73-item rating scale and consists of ten scales. Nine scales measure unpleasant effects: weight gain, sexual anhedonia, sedation, affective flattening, EPS, reduced sociability, increased sleep and total unpleasant effects. The last scale added the unpleasant scales, including the remaining items. The recovery scale measures the enjoyable responses attributed to the antipsychotics.
- Internalized Stigma of Mental Illness [55] (ISMI). Sub scales: alienation, stereotype endorsement, discrimination experience, social withdrawal and stigma resistance. Good internal consistency, test-retest reliability and construct validity.
- Self-esteem Rating Scale- Short form [56] (SERS-S)
- Psychosis Attachment Measure [57] (PAM) is a 16-item self-rating scale measuring attachment anxiety and attachment avoidance. This may be an important predictor of symptoms, interpersonal problems and difficulties in therapeutic relationships over and above severity of illness.
- Positive and Negative Syndrome Scale [33] (PANSS): this is a 30-item rating scale designed to measure severity of psychopathology in adult patients with schizophrenia. Five components have been reported: positive, negative, depression, agitation-excitement, and disorganisation.
- Global functioning will be assessed using the GAF questionnaire [32].
- The Premorbid Adjustment Scale [58] (PAS) is a rating scale about five domains of functioning: sociability, peer relationships, scholastic performance, adaptation to school and social-sexual aspects. It covers two life periods: up to 12 and 12 to 16.
- The Recovery Assessment Scale [37] (RAS): this is a 30-item rating scale designed to measure recovery.
- The Multidimensional Scale of Perceived Social Support [59] (MSPSS) is a 12-item instrument that measures perceived social support. Four scales have been reported: significant other, family, friends and total scale.
- Aggressive and suicidal behavior will be evaluated using a self-composed list of self-report questions (developed at the department of Psychiatry, UMC Utrecht).
- Cigarette, alcohol and drug abuse (as assessed with the alcohol and drug section of the CASH [38] and the WHO assist [60].
- The MINI-Screener is a self-report questionnaire and consists of fife sections: generalized anxiety disorder, panic disorder, social anxiety disorder, obsessive compulsive disorder and post-traumatic stress disorder (developed at the department of Psychiatry, UMC Groningen).
- Childhood trauma will be evaluated by asking subjects to fill-in a retrospective self-report questionnaire, the Childhood Trauma Questionnaire-Short Form [39] (CTQ-SF).
- Language production will be assessed by an automatic analysis of spoken language. Patients will be asked to talk for approximately five minutes, with a maximum of ten minutes. The recordings will be automatically parsed and annotated using computer learning language systems.

### Cognitive assessment

Neurocognitive functioning will be assessed with the Brief Assessment of Cognition [31] (BACS) and the Stroop task [61]. The BACS is derived from larger neuropsychological batteries and provides a valid estimate of general cognitive ability. The BACS consists of the following seven subtasks:

- *Verbal memory - List learning:* Patients are presented with 15 words and then asked to recall as many as possible, which will be repeated five times. Measure: number of words recalled per trial, in any order.
- *Working memory - digit sequencing task:* Patients are presented with clusters of numbers of increasing length and are asked to tell them in order from lowest to the highest. Measure: number of correct responses.
- *Motor speed - Token motor task:* Patient are given 100 tokens and are asked to place them in a container as quickly as possible. Measure: number of tokens correctly placed into the container.
- *Verbal fluency - Category instances:* Patients are asked to name as many words in a certain category in 60 s (supermarket items, tools). Measure: number of unique and appropriate answers per category.
- *Verbal fluency - Controlled oral word association test:* Patients are asked to generate as many words as possible that begin with a given letter in 60 s. Measure: number of unique and appropriate answers per category.
- *Attention and speed of information processing - Symbol coding:* Timed paper-and-pencil test in which respondent uses a key to write digits that correspond to nonsense symbols. A sheet with a 9 item key is provided, pairing digit 1–9 with a unique symbol; below are rows of numbers with blank squares beneath. The subject pairs each number with its unique symbol. Measure: number of correct number-symbol pairs completed in 90 s.
- *Executive functions - Tower of London:* Patients are shown two pictures simultaneously with 3 pegs uniquely arranged in each picture. Patients are asked to give the number of times the balls in one picture need to be moved in order to make the arrangements identical on both pictures. There are 20 trials in with variable difficulty. Measure: number of correct answers.

In addition, the Stroop task will be administered, which measures executive functioning (verbal inhibition). Patients are shown three cards on which words are depicted in a 10 × 10 matrix. Card 1 contains color words (red, blue, yellow and green) that are printed in black ink in random order. Card 2 displays solid color patches in one of these four basic colors. Card 3 again contains color words, but these are printed in an incongruous ink color. Individuals were instructed to read the words (card 1), name the colors (card 2) and, finally, name the ink color of the printed words (card 3) in three subsequent sessions. Measure: reaction time and number of errors.

### Movement disorders

Patients are examined for movement disorders during baseline, the close-out visit (at six months) and during each follow-up visit. A standard protocol will be used, as described by Van Harten and colleagues [62]. Patients are barefooted and seated in a chair without armrests. The researcher asks detailed questions about (i) use of chewing gum or candy at the moment of assessment as well as (ill-fitting) dentures, as both may be misdiagnosed as orofacial movement disorders, and (ii) subjective akathisia. The patient performs different tasks to assess the existence of movement disorders and to provoke abnormal movements. Thus, the following positions are adopted in succession: resting arms on the lap in different positions, arms hanging aside, stretching arms, making fast alternating hand and foot movements, opening the mouth, showing the tongue, rising from chair, and walking. Additionally, posture, rigidity and balance are assessed. Tongue dyskinesia is provoked by fingertip movements, and objective akathisia by talking conversationally while the patient is standing.

- Dyskinesia (American Psychiatric Association, 1992) is defined as hyperkinetic choreiform involuntary movements which often fluctuates in severity. Tardive dyskinesia is assessed with the St. Hans Rating Scale [35] (SHRS) and case definition is based on Schooler and Kane criteria [63], requiring (i) the presence of moderate dyskinesia in at least one body area or mild dyskinesia in at least two body parts, and (ii) the absence of other conditions resulting in abnormal involuntary movements.
- Parkinsonism is also assessed with the SHRS [35]. A case definition of parkinsonism is based on (i) mild expression of rest-tremor or rigidity as both are typical of parkinsonism, and (ii) if no tremor or rigidity is rated, the cut-off point is one rating of moderate or two ratings of mild on items of bradykinesia and postural stability. The more stringent criteria for items of bradykinesia and postural stability are chosen as these symptoms may be part of psychiatric syndromes or sedation.
- Dystonia is defined as a syndrome of sustained muscle contraction, frequently causing twisting and repetitive movements or abnormal postures [62]. Tardive dystonia is diagnosed, following Burke’s criteria [64], if one body area attracted a rating of at least mild or if two or more body areas attracted a rating of slight on the SHRS [35]. As frequent eye-blinking (rating of ‘mild’ on the item ‘eye’) has many causes, case definition of tardive dystonia required a rating of at least moderate (blepharospasm) when ‘eye’ is the only symptom area.
- Akathisia [65] is defined as both subjective inner feelings of restlessness and objective motor (leg) movements. A case definition of akathisia is based on a rating of at least ‘mild’ on the global akathisia item. Akathisia is assessed with the Barnes Akathisia Rating Scale [36] (BARS) comprising an objective and a subjective item.

### Additional measures

- Weight will be measured during each visit. In addition, abdominal and hip circumference, height and body mass index (BMI) will be noted. Blood pressure and pulse will be determined. This way, presence and severity of metabolic syndrome can be evaluated, as defined by the American Heart Association/National Heart, Lung and Blood Institute [66] (AHA/NHLB).
- Medication use will be listed in a table, evaluating type of medication, reason for use, dosage (average dose per period), start and stop date, reason for stopping/switching of medication.
- Use of psychoactive substances will be recorded. Frequency, quantity of use and start date of alcohol and cannabis will be scored. For other psychoactive substances only frequency will be scored.
- The number of hospitalizations and relapses are also taken from the patient’s file. Using information of the pharmacy, the mean cumulative dose of antipsychotic medication taken since baseline is calculated. Relapse is defined as clinical deterioration during at least 1 week, having consequences (augmentation of antipsychotic dosage, hospital admission, or more frequent consultations), reported by the clinician and subsequently confirmed by PANSS positive subscale item scores assessed by a research team member, of at least one score of 5 (moderately severe) [33]. In contact with justice: number of times is noted.
- Involuntary admission for treatment: number of times and duration is noted.

### BeHapp

#### General description

BeHapp is a smartphone application which is developed for the direct day-to-day registration of several aspects of daily life concerning the behavior of humans. The application continuously monitors communication and exploration patterns in patients and controls as a function of social acts, environment social density measurements, GPS location updates and general smartphone usage. This method circumvents the issue of subjectivity given that no active input is required from the participant, other than installing the application and providing some baseline information.

#### Methods

The application, once installed and initialized, passively collects (meta-) data about phone call activity, bluetooth devices and WiFi access points in the direct vicinity of the participant, location updates and mobile application usage. By means of integrating this data a multidimensional behavioral profile of the participating individuals can be obtained.

The application is currently only compatible with the Android platform. Each participant will be asked if they use an Android smartphone and if they consent to this part of the study. After thoroughly explaining what the application does, the participant will be provided with an e-mail message containing instructions on how to obtain and initialize the application. Initialization consists of entering a (single-use) short-key to identify oneself to the application, this key is also provided in the aforementioned e-mail message. If preferred by the participant this process can be completed with our help as well.

#### Privacy

Several measures are taken so as to protect the privacy of our participants:

- As a base principle, no directly identifiable information about any participant is stored within any system that is part of the BeHapp service. Participants are represented by unique id’s, which are associated with identifiable information in decentralised third party information systems.
- In order to decrease the attack potential (‘attack surface’) of the BeHapp service, the publically accessible centralised software components are designed and implemented to work with least privileges and least programming logic. By this we aim to prevent retrieval of participant data through the application layer of our service.
- Monitoring data is encrypted before being stored locally on the device of a participant and after each successful upload, all of the local monitoring data is cleared from the device. Bother measures are in place to maximally reduce risk of revealing privacy-sensitive information to third parties in case of loss or theft of the smartphone.
- All communication streams between the mobile applications and our centralized data storage components will go through secured (encrypted) channels using modern industry standards.
- Information gathered about identities of third persons, e.g. contacts of participants are obfuscated on the phone before being sent to the central data store. Obfuscation is performed using so called one-way-hashing / encryption techniques. This allows the researcher to determine whether the same instance (person or device) has been recorded more than once while preventing them from directly identifying the recorded instance.
- The BeHapp service resides within the Google Cloud Platform which employs a security model built on 15 years of experience in protecting customer data. The platform is compliant with HIPAA, ISO 27001 and the EU Data Protection Directive. Furthermore, all data stored is encrypted by default ensuring that only researchers part of the BeHapp team are able to access the data.
- In order to reduce the attack potential (‘attack surface’) of the BeHapp service, the publically accessible centralised software components are designed and implemented to work with least privileges and least programming logic. By this we aim to prevent retrieval of participant data through the application layer of our service.

#### Duration of measurement

The duration of measurement, i.e. the time that BeHapp actively collects and uploads data on the participant, is variable and may depend on the specific strategy and aims of the study it is employed in. At any time during the course of the study you can decide to stop BeHapp from collecting information by stopping the application in the application manager (and re-activating it when desired) or removing the application permanently from your device.

## Abbreviations

AE: Adverse event
BACS: Brief Assessment of Cognition in Schizophrenia
BARS: Barnes Akathisia Rating Scale
BMI: Body mass index
CASH: Comprehensive Assessment of Symptoms and History
CRF: Case report form
CTQ-SF: Childhood Trauma Questionnaire Short Form
DI: Dedicated includer
DSM-5: Diagnostic and Statistical Manual of Mental Disorders
eCRF: Electronic case report form
EMA: Ecological momentary assessments
EWS: Early warning signs
FEP: First episode of psychosis
GAF: Global assessment of function
GCP: Good Clinical Practice
HAMLETT: Handling Antipsychotic Medication Long-term Evaluation of Targeted Treatment
MINI: Mini International Neuropsychiatric Interview
MRI: Magnetic resonance imaging
PANSS: Positive and Negative Syndrome Scale
PET: Positron emission tomography
PI: Principle investigator
RAS: Recovery Assessment Scale
SAE: Serious adverse event
SD: Standard deviation
SHRS: St. Hans Rating Scale
SPIRIT: Standard Protocol Items Recommendations for Interventional Trials
STG: Superior temporal gyrus
UMCG: University Medical Center Groningen
WHO-DAS 2.0: World Health Organization Disability Assessment Schedule
